# Combining Integrated Informative System and Historical Digital Twin for Maintenance and Preservation of Artistic Assets

**DOI:** 10.3390/s21175956

**Published:** 2021-09-05

**Authors:** Adriana Marra, Salvatore Gerbino, Alessandro Greco, Giovanni Fabbrocino

**Affiliations:** 1 Institute for Construction Technologies, Italian National Research Council, ITC-CNR, 67100 L’Aquila, Italy; marra@itc.cnr.it; 2 Department of Engineering—University of Campania “Luigi Vanvitelli”, 81031 Aversa, Italy; salvatore.gerbino@unicampania.it (S.G.); alessandro.greco@unicampania.it (A.G.); 3 Department of Biosciences and Territory, University of Molise, 86100 Campobasso, Italy

**Keywords:** cultural heritage, laser scanning, conservation, digital twin, maintenance, 3D simulation

## Abstract

The protection of artistic and cultural heritage is a major challenge due to its peculiarities and its exposure to significant natural hazards. Several methodologies exist to assess the condition of artistic heritage and to protect it from exceptional actions. Moreover, novel digital technologies offer many solutions able to deliver a digital replica of artifacts of interest, so that a reduction in the uncertainties in the analysis models can be achieved. A rational approach to the preservation and protection of artistic heritage is based on traditional approaches supported and integrated by novel technologies, so that qualitative and quantitative indicators of the current condition of artistic heritage can be defined and validated in an interdisciplinary framework. The present paper reports the results of an approach to the maintenance and preservation of art objects housed in a museum complex based on a comprehensive digital path towards a Historical Digital Twin (HDT). A workflow aimed at estimating the stress regime and the dynamic properties of two sculptures, based on the detailed three-dimensional model resulting from a laser scanner survey, is illustrated and discussed. The results highlight the great advantages resulting from the integration of traditional and novel procedures in the field of conservation of artistic assets.

## 1. Introduction

The need to protect cultural heritage from natural and manmade risks has led to increased attention on the issues of its conservation and assessment of current condition. The safeguarding of cultural heritage is a difficult challenge for several Mediterranean countries that are located in high seismicity areas and host a large heritage to be preserved. This heritage includes magnificent architectural works, artistic objects displayed in museums, and valuable assets, such as stuccoes, frescoes, sculptures, etc., that are an integral part of architectural heritage.

The damage caused by major seismic events has highlighted the need to prevent risks and protect architectural and artistic assets. However, these goals are particularly complex to achieve due to the peculiarities of this cultural evidence and the interactions existing between structural and non-structural components, namely between structures and artistic assets. Several international [1,2,3] and national [4] recommendations have been approved to face these issues and identify proper methodologies for the knowledge and analyses of cultural heritage.

These recommendations aim to support the assessment of safety and conservation of cultural assets and the identification of interventions to be implemented for their protection and valorization. The proposed approach requires the integrated action of different skills to achieve a high level of knowledge about the analyzed artifact and to identify structural interventions which are able to consider the peculiarities of historical assets. The knowledge path allows us to minimize uncertainties in the structural and seismic assessment of cultural heritage based on the integration between historical analysis and careful investigation on geometry, materials, and state of degradation.

The technological advances in the architectural field, in particular for the metric surveys [5,6,7], offer great advantages as they reduce errors in geometry [8] and provide results useful for documentation, knowledge [9,10], conservation, and valorization [11,12]. The photogrammetric survey and laser scanning are the most common approaches used for this purpose. They enable the acquisition of semantic and spatial data [13,14] and the development of a high-precision three-dimensional model that can be used to share heterogeneous information [15] and analyze structural performance [16]. At the same time, novel analysis methods for assessing the vulnerability of historical [17,18,19] and artistic heritage [20,21,22] have been developed to mitigate the risk of loss in case of an earthquake. However, a systematic approach based on the correlation of traditional and innovative tools, and aimed at the knowledge and management of movable heritage concerning natural disasters mitigation, is not adequately shared [23].

Therefore, novel approaches and tools should be defined to overcome the uncertainties concerning the typological, material, and constructive peculiarities and achieve a comprehensive knowledge of artistic assets. In such a context, the digital models derived from geometric surveys and three-dimensional modeling provide useful support to manage and exchange heterogeneous data acquired from the analyses carried out.

The correlation between qualitative and quantitative information, namely between traditional approaches and innovative ones based on digital technologies, encourages the development of Integrated Informative Systems (IISs) that facilitate the preservation and valorization of cultural heritage [24,25,26].

These informative systems support the conservation, maintenance, and management processes identifying when and how to intervene. Moreover, the development of such systems towards the Historical Digital Twin [27,28] will provide the proper strategies to be implemented, starting from the analyses and simulations performed on digital replicas, and hence from the priorities identified by the system.

In the following, some basic features of the IIS—relevant from the technical and scientific standpoint—are investigated and validated by means of the real application to some art objects housed in the Archaeological Museum of the Ancient Capua, located in the municipality of Santa Maria Capua Vetere, South Italy. Qualitative and quantitative analyses were performed starting from the three-dimensional models of art objects to assess their state of conservation and their seismic response and damage mechanism. The obtained results are the key step of the IIS, and they highlight the advantage of using digital technologies in the process of knowledge and conservation of artistic assets.

The paper, which follows and extends the outcomes of the study [29], is organized as follows. Section 2 provides the approach and tools used to develop the Integrated Informative System via the reference of real cases; Section 3 provides the results of analyses carried out; and, finally, Section 4 reports the conclusions.

## 2. Materials and Methods

This section reports methods and tools at the base of the approach proposed to define reliable and effective design specifications of Integrated Informative System (IIS) and Historic Digital Twin (HDT). The topic is addressed with reference to two statues, displayed in the Archaeological Museum of the Ancient Capua, selected as they are different from one another from a historical and material point of view, and each have a high symbolic and cultural value. These features make the artistic assets a relevant case study for the interpretation of the analysis results and the future implementation of the IIS and HDT.

### 2.1. Methodological Approach and Survey Tools

**Figure 1:**
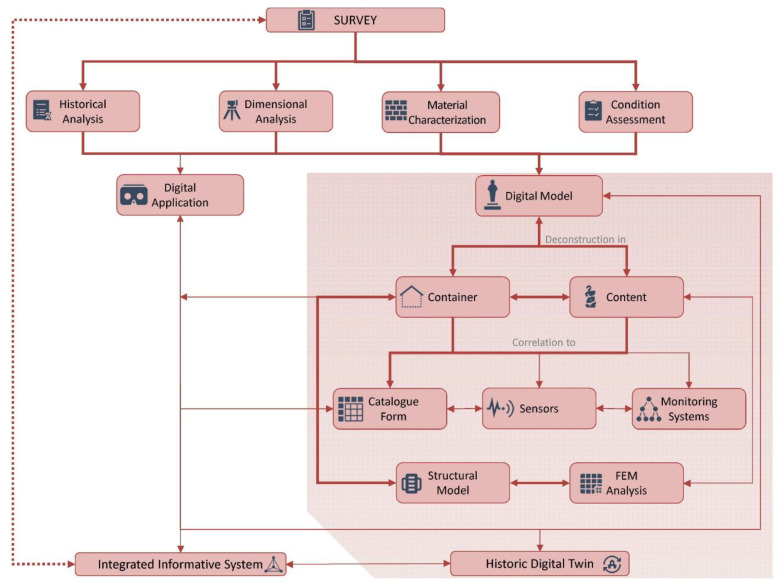
General framework of the research workflow.

The safeguard and conservation of artistic heritage is a complex task as several features should be analyzed, and as strong interrelation and interaction exist between the artworks, or content, and the container asset, which is the building and the structural elements that ensure its stability. Integrated analysis methods for the knowledge, assessment, and management of these assets should be defined, despite the efforts made in developing novel analysis methods and the high-precision tools that support the preservation of movable heritage. The methodology proposed in the research is based on interdisciplinary and systemic approach, according to the international recommendations and the principle of conservation and safeguarding of historic buildings [2,4,30]. The approach is divided into different phases through which it is possible to achieve a comprehensive knowledge of the artifacts, obtaining an overall and integrated view. The process, aimed at improving conservation and safety by means of specific technical and scientific activities, is illustrated in Figure 1. It is worth noting that the general framework of the process is described by using different line types, which differentiate those phases tested and validated during the experimental program (thick red line) from those designed but not yet executed (thin red lines) and those that need further work to be optimized and applied under real operational conditions (dotted red line).

The key phase of the process is represented by the integrated survey of the artefact, which is divided into four essential interconnected moments [31]. The first consists of the historical, artistic, and architectural analysis of the artifact. The historical specificities of the artifact, in terms of temporal transformations, materials, and construction techniques, are identified through these analyses. As a result, a detailed chronological report of the published and available sources can be drawn up, summarizing the history, the transformations, the restorations, or the partial destructions that have affected the artifact. At the same time, significant information on the mechanical properties of the materials can be derived from a detailed cross-sectional analysis of documentary and bibliographic sources. This information is generally qualitative, but can be converted into quantitative data if appropriately compared with the extensive technical and normative literature, which provides the mechanical parameters of the materials identified for performance assessments.

Another relevant moment of the process is the acquisition of the dimensional data of the artifact under investigation, recurring to direct or indirect metric survey activities. A two- and three-dimensional representation of the asset, as a whole and in its different elements, is obtained as a result of the metric survey. These results represent the object in the conditions in which it appears at the time of the metric survey, making a useful support for the graphical representation of the information acquired during the other investigations. Indeed, each reproduced element can be correlated to the information concerning the history, materials, and state of conservation. The latter considerations, in particular, can be performed on high-resolution photo plans derived from the photogrammetric survey processes.

Finally, an initial diagnosis of the current state can be made through the combination, correlation, and representation of all this information. In such a way, the research can be directed towards other fields of investigation to complete the knowledge framework defined. Additional information can be collected from field or laboratory investigations that reveal hidden points in the history of the artifact investigated [32].

The results of the survey process, summarized in Table 1, can be organized into digital applications [33,34], which are outside the scope of this paper, and three-dimensional models, which are created after the interpretation and decomposition of the object into container, content, and sensors. Through this type of modeling, it is possible to improve the management and cataloging of the information collected and connect the model to the monitoring systems. On the other hand, the decomposition of historical artifacts’ elements into digital modeling is also valuable in the assessment of structural performance, as it is possible to evaluate the response of all component elements in a singular manner or jointly.

Finally, the heterogeneous information collected during the interdisciplinary analysis should be included in appropriate relational databases or other existing systems (e.g., structural and environmental monitoring systems, moisture, light, or temperature detection systems) related to the digital model of the artifact.

The aim of this procedure, which integrates traditional and innovative methods and tools, is the development of an Integrated Information System (IIS) [24]. This system exploits the potential of Information and Communication Technologies (ICT) in managing information acquired in a coherent and coordinated manner, improving the data’s availability to those in charge of safeguarding heritage or to the specialists involved in the analysis and protection processes. The implementation of the IIS and its correlation to dynamic data, namely the monitoring systems linked to the IIS and the automated processing procedure of the information collected in the system, provides the basis for the development of the Historic Digital Twin (HDT). The HDT is conceived as a digital replica (digital twin) of the main historical, geometric, mechanical, and structural characteristics of a physical object that, thanks to the mutual relationship with other data acquisition and management systems, Internet of Things (IoT) based technologies, and Machine and Deep Learning applications, has the potential to change the conservation, management, and maintenance processes of the historical and cultural heritage [35].

### 2.2. The Real World Test Cases at the Archaeological Museum of the Ancient Capua

**Figure 2:**
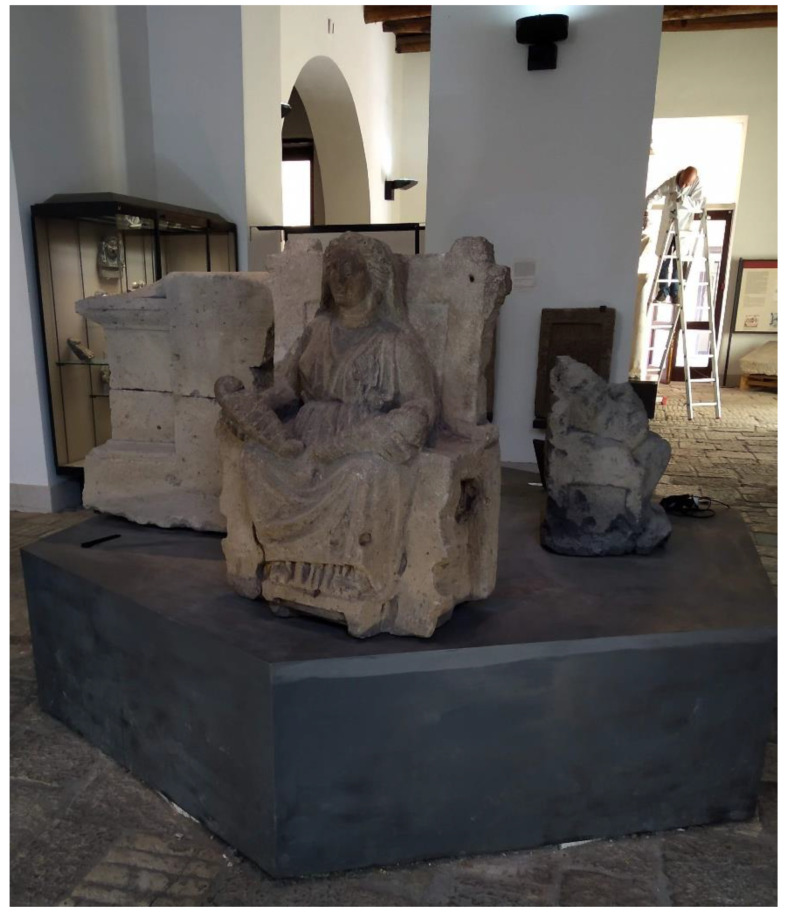
The *Mater Matuta*, Archaeological Museum of the Ancient Capua.

**Figure 3:**
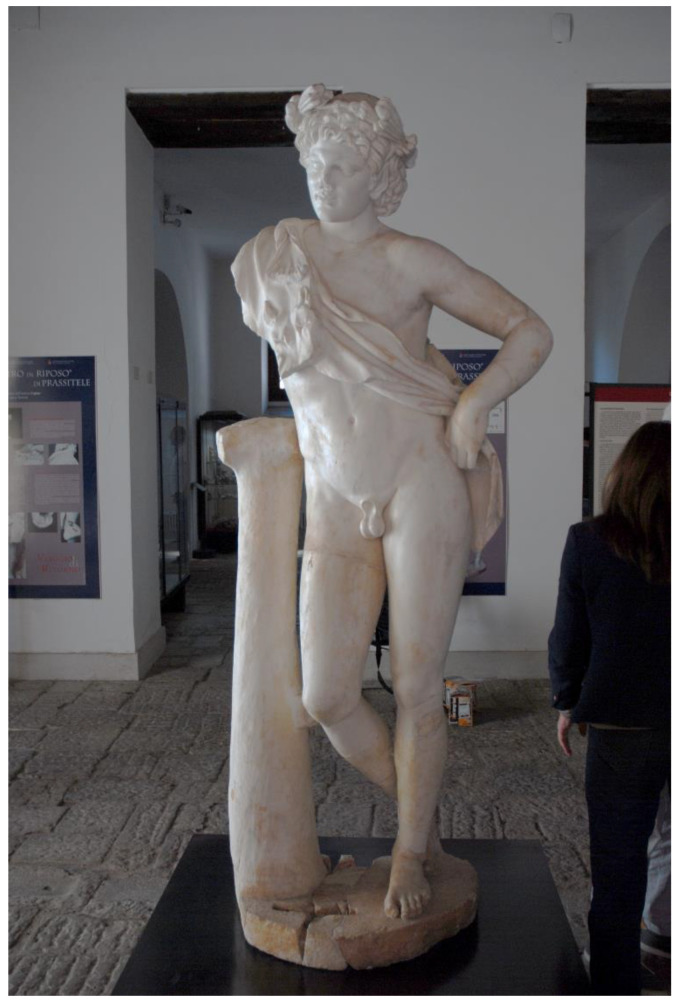
The *Resting Satyr*, Archaeological Museum of the Ancient Capua.

The basic concepts and some operational phases of the workflow described in the previous section were tested for the sake of validation on a real case, two artworks exhibited in the Archaeological Museum of the Ancient Capua. The museum is housed in a 19th century building that, in the past, included the medieval tower of St. Erasmus, built on the ancient roman Capitolium [36]. The museum was created to provide a modern display of materials found during the excavation activities carried out in the second half of the 20th century in the area of the ancient Capua [37]. Among the artworks housed in the Museum, the *Mater Matuta* (Figure 2) and the *Resting Satyr* (Figure 3) have been analyzed, as they have a high historical and cultural value.

In the first phase of the survey, the information concerning the history of the container and content, their dimensional and material features, their construction techniques, and their degradation and alteration state have been acquired. In this phase, traditional tools have been used, such as bibliographic and iconographic sources and cataloging tools, to record all information acquired or derived from direct analysis.

The metric survey of artworks has been carried out by 3D scanning with laser technology using a high-performance device, the HandyScan 700 by Creaform [38], which is not designed to detect and store data associated to the color of the scanned object. This is the reason why a photogrammetric survey session has been performed in a way that a good quality texture of the surface was acquired, and high definition orthomosaics processed to support the analysis on the state of conservation of the artifacts at the time of the survey.

In the following phase, the information collected during survey and knowledge analyses has been digitalized within the Seismic Vulnerability Assessment of Movable Heritage—SeVAMH—form [39]. This tool can record and share heterogeneous information about the container and content, and assess the state of conservation, the main seismic response, and damage mechanisms of movable heritage in a qualitative manner [40].

The correlation between quantitative data, derived from the metric survey and implemented analyses, and qualitative data, summarized in the SeVAMH form, makes it possible to achieve extensive knowledge of the investigated assets. Such information is useful in the definition of possible diagnostic investigation as well as in the planning of structural monitoring. These activities would provide details on materials of the artifacts and on the quantitative assessment and monitoring of their state of conservation.

A further phase is the application of the survey to the structural components of museum buildings. These analyses would give a better description of the relationship existing between the building and artistic heritage, in a view of container and content. Therefore, it is possible to identify structural models to be used in the assessment of current conditions for maintenance and restoration purposes as well as in the quantitative assessment of the effects generated by major hazards to which these assets are exposed, for instance the seismic one [16,41].

In this way, an Integrated Informative System, i.e., the static part of the HDT, was defined. This system represents, as previously explained, a suitable tool not only for coordinating the share and management of information, but also for the safeguarding and valorization of cultural heritage [35,42]. The main goal of the IIS is to reduce the time and costs of conservation and restoration processes, thanks to the availability of variously interconnected heterogeneous information, and at the same time ensuring the safety of the movable heritage exhibited in museums against natural hazards. In addition, the IIS represents a tool to support the development of the Historic Digital Twin [35] for whose practical implementation it is necessary to define the procedures for the management, integration, and automatic processing of the collected big data.

### 2.3. Movable Asset Survey Session

An experimental acquisition session was set up at the Archaeological Museum of the Ancient Capua in order to collect data necessary for the 3D reconstruction of the two statues: *Mater Matuta* (Figure 2) and *Resting Satyr* (Figure 3).

As already mentioned, laser scanning technique was used to achieve a metrically accurate three-dimensional model to be used for detailed analyses assessing the safety of artworks, while the photogrammetric study was implemented in order to deploy a photorealistic representation to improve the detail of those aspects of the survey associated with the conservation and valorization of movable cultural heritage.

**Figure 4:**
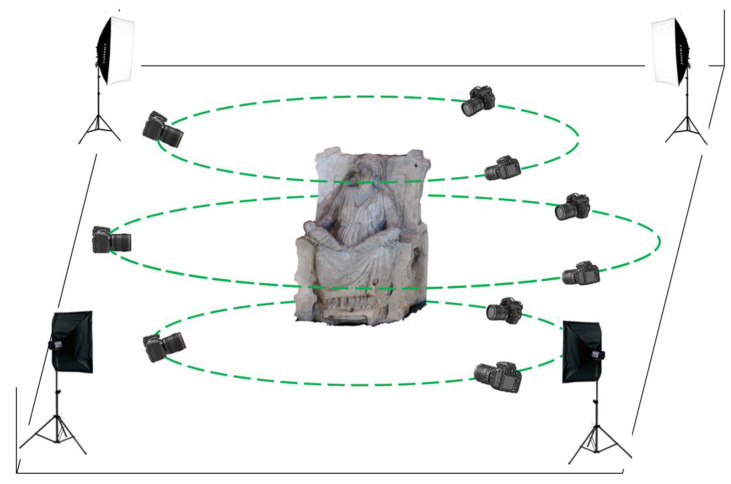
Layout of the photogrammetric acquisition area.

The complete survey protocol was tested and validated in the case of the *Mater Matuta*. The photogrammetric acquisition area was set up to ensure a good rendering of the images using 4 lighting boxes, totaling 720 W of LED light at 5500 K temperature, placed at the corners of the acquisition area, see the sketch reported in Figure 4.

High resolution photographs (7360 × 4912 px sized) were captured, with constant focus, at 3 different heights rotating 360° around the object by means of a NIKON D810 (36.3 Mpx) professional digital camera equipped with a 35.9 × 24 mm sized sensor. The camera was calibrated by means of Matlab^®^ Camera Calibration Toolkit [43] independently from the software used to process the images for the photogrammetric reconstruction. Table 2 resumes the parameters of the photogrammetric acquisition; average values of the heights and angles of acquisition are reported.

In the scene, a reference bar was also positioned to be able to scale the reconstructed model with texture to be then fit to the scanned laser model.

Once the photogrammetric acquisition was completed, the statue was covered with about 200 small circular targets for laser acquisition, as requested by the handheld laser scanner, HandyScan 700 by Creaform [38]. This metrological device acquires high-quality measurement data (device accuracy up to 0.03 mm and measuring speed of at least 480 k points/s according to the Creaform product sheet). Targets work as three-dimensional references for the free handheld scanner to allow it to be moved all around the object.

The process of laser acquisition of the *Resting Satyr* was completely similar.

The photos collected during the photogrammetry acquisitions were processed by using Metashape Professional by Agisoft^®^ [44] software environment. The steps followed to generate the final 3D mesh model is described in Section 3.1.

## 3. Results

The present section reports, after the assessment of the seismic hazard at the site, all the data and the information associated with the acquisition and interpretation of historical documents and data following the historical research on the assets and the technical operations on the two statues. This way of reporting the study is intended as a more effective and clear definition of the nature and the potential use of technical and historical data in view of a well-balanced and design archiving process and definition in the prospective IIS.

### 3.1. Assessment of the Seismic Hazard at the Site

The municipality of Santa Maria Capua Vetere is located in the Agro Casertano area, southwest of Mount Tifata, south Italy, and lies in a sub-flat area with an extension of about 14 km^2^. Different sources provide an overview of the geological and geotechnical characterization of the municipality, along with relevant technical data in view of the basic characteristics of the area from a seismic perspective [45].

The available data report widespread soils with variable thicknesses of pozzolana, pumice, and stratified lapilli, related to the volcanic phenomena of the Campi Flegrei. Therefore, the local stratigraphy reveals layers of silty-clay pozzolana and sandy levels with big pumices and scoriae, below which there are sandy levels of pyroclastic origin with a thickness and lithic variable. In the deeper layers, sandy silt and clay levels are found for a thickness of 10–15 m beyond the 40–50 m elevation; in the southern part of the area, the geological analysis revealed a tuff, yellow or grey, with numerous black slags and a variable thickness of 3–6 m. The low slope of the ground hinders the development of both deep and superficial gravitational phenomena; however, the subsoil equilibrium conditions are altered by the presence of anthropic and natural caves, which have not been sufficiently surveyed and have caused collapses in some areas of the municipality in the past.

It is worth noting that many in situ investigations are found in the technical literature so that the geological and geotechnical model of the municipality of Santa Maria Capua Vetere can be used to identify the different homogeneous areas of the territory in terms of characteristics and seismic response [45,46].

**Figure 5:**
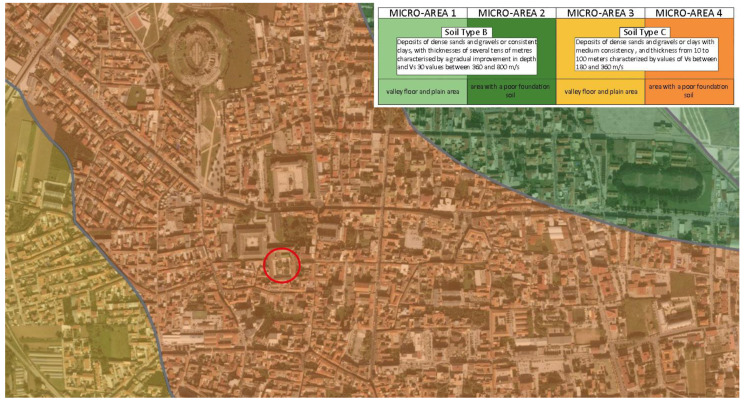
Map of homogeneous areas zoning in seismic perspective: the red circle identifies the location of the Archaeological Museum of the Ancient Capua.

The results of the abovementioned investigation have enabled the development of the seismic zonation map of the city, from which is clear that the Archeological Museum of the Ancient Capua is located in a flat area with a poor foundation soil (Figure 5). Therefore, the museum area is characterized by a type C foundation soil, namely “deposits of dense sands and gravels or clays with a medium consistency, and thickness from 10 to 100 m characterized by values of vs. between 180 and 360 m/s” [47].

Based on the information reported above, the seismic hazard at the site can be evaluated according to the Italian civil engineering structures design code for the Type C soil [48]. Table 3 reports the main parameters associated with the definition of the inertial forces determined by the ground shaking. In particular, the site horizontal acceleration expected at the bedrock level, a_g,H_, is provided depending on the return period, T_R_, moving from very frequent events—T_R_ = 30 years—to very rare ones—T_R_ = 2475 years. The spectral acceleration computed for rigid structures—T = 0—are also defined for both horizontal—S_a,H_(T)—and vertical—S_a,V_(T)–directions. It is worth noting that the design seismic actions are not dependent upon the structural features of the building, being the exhibition located on the ground floor and the building characterized by a single-story.

### 3.2. The Mater Matuta

**Figure 6:**
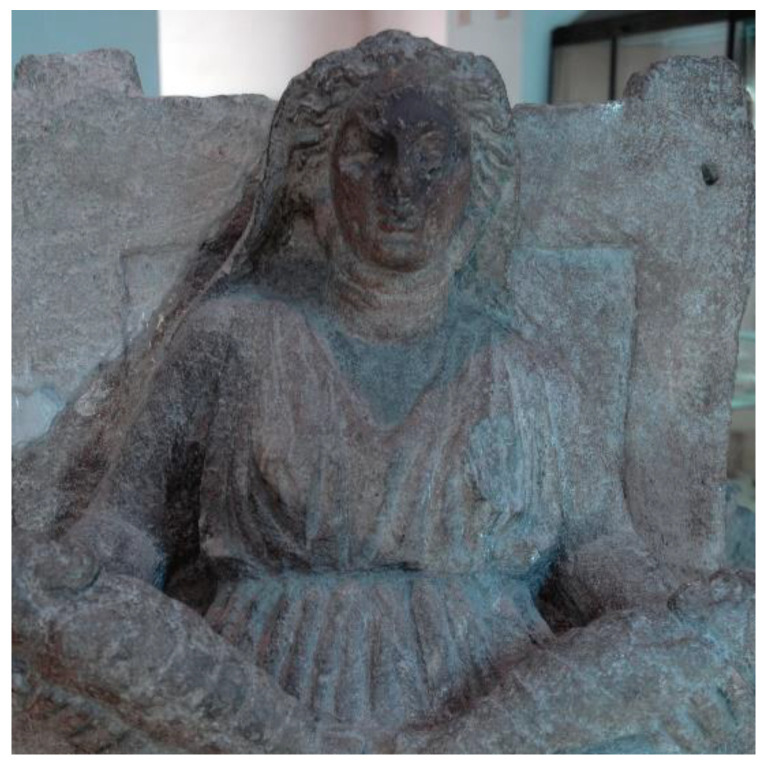
The *Mater Matuta*, details.

The analysis on the bibliographic sources revealed that the statue of *Mater Matuta* (Figure 6) is one of the finds discovered during building works carried out in 1845 by Carlo Patturelli on a site located in the Petrara area along the ancient via Appia, outside the ancient town of Capua and near a gate traditionally dedicated to Juno [49]. The remains of a sacred building and several statues, which depict female figures seated on thrones holding one or more infants, were found during this work. The finds were quickly reburied, and the excavation activities were resumed only in 1873 “with the aim of selling the most beautiful and valuable artifacts on Italian and European art markets” [49] (p.120). Several finds, such as many *Matres Matutae*, were transferred in 1876 to the Campano Museum, founded in 1870 and opened four years later [50]. The high number of *Matres Matutae* statues found in the area are evidence of the presence of a temple for the worship of the Italic goddess of dawn and birth (*Mater Matuta*), and the statues are an offering and thanksgiving for the gift of fertility and maternity [50]. One of the *Matres Matutae* displayed in the Archaeological Museum of the Ancient Capua has been analyzed in detail.

The statue of *Mater Matuta*, according to the analyses carried out by Nava [49] on the *Maters* of Campano Museum, is probably one of the mothers that combine features of Italic and Greek art, and it dates to the 4th and 3rd centuries B.C. The statue is in grey tuff from Mount Tifata; it shows a strong symmetry in figure construction and dysmetria in the legs position. The forms appear essential, and the surfaces are carefully worked. The head is slightly rotated to the right; the body is wrapped in soft and non-symmetrical drapery. The figure is enriched by other elements: the veil that covers the head and the earrings. The infants are placed on the *Mater*’s lap on not-aligned plans and are wrapped in straps. Finally, the throne on which the figure is seated has a high back and molded legs.

The two-step survey was implemented as follows: (i) the photogrammetry, in order to obtain detailed information concerning the state of conservation and present condition of the asset and make available for further developments of the geometrical model a reliable texture for the scanned model; (ii) the laser scanning phase, to acquire the main geometric features of the statue. As the laser scanning phase is concerned, about 200 circular markers were put on the statue and on the support plane, around its immediate surroundings. These small markers are automatically recognized by the scanner and are used as local references during the acquisition phase, namely when the scanner moves all around the object (dynamic referencing).

**Figure 7:**
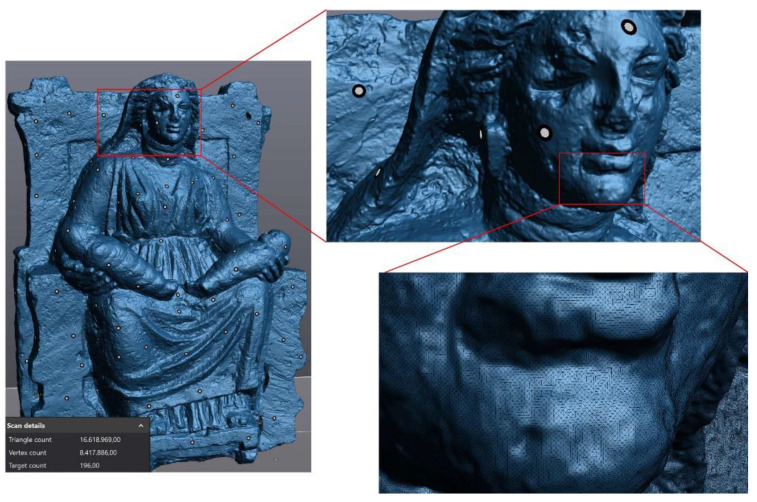
Result of the laser scanning process.

The accuracy of the scanning process [51] is strictly related to the provided calibration table for the selected device. This test is made once before running any new measurement task. The accuracy changes according to the calibration certificate as 0.020 + 0.060 mm/m. All the recommendations in using the scanner (as suggested by the manufacturer to achieve the best results) were adopted. Examples are the orientation of the scanner with respect to the object and the feature to be captured, the speed of movements, the average distance between markers, etc. After scanning the statue, the acquired mesh model was post-processed (in VXelements by Creaform [38]) removing outliers, reducing noise, filling small holes, reconstructing the base plane to obtain a closed model, and re-meshing the whole model to make the mesh elements more uniform and regular, but keeping the same original accuracy (average nodes spacing about 0.05 mm). Figure 7 shows the result of the 3D laser scanning process where the mesh model has a high level of detail due to the scanner precision. The final cleaned mesh model, after the post-processing phase, counts more than 8.4 M vertices and 16.5 M triangular elements, starting from the original data set with more than 17.3 M elements. The final model is 734.5 mm wide, 439.5 mm deep, and 991.5 mm high.

**Figure 8:**
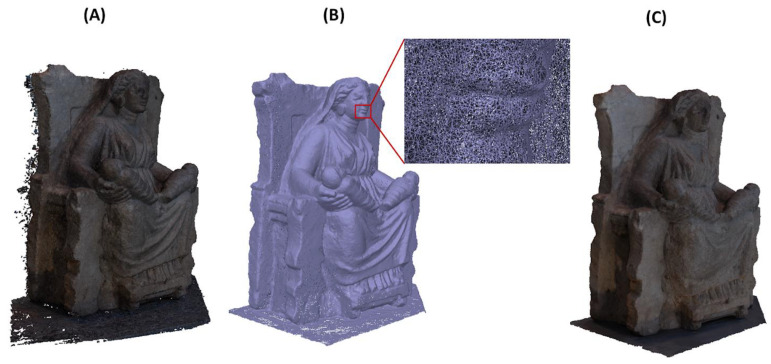
Initial dense point cloud model (**A**), mesh model (**B**), and textured model (**C**) resulting from photogrammetry after the mesh editing phase.

In order to obtain a reliable documentation of the current state of the asset [6,52], 120 photos were taken all around the statue, discarding blurred or low-quality photos. Only 98 photos were processed by Metashape Professional (Agisoft) software, reconstructing a model characterized by about 127 k triangles and 63 k vertices (Figure 8). The editing of the acquired data set is always necessary to clean the model by reducing noise, eliminating extra objects eventually captured in the scene, filling holes, and remeshing the model for a better rearrangement of the mesh without altering the geometrical features. It is worth noting that software tools providing an automatic procedure to clean the whole model exist, but it is suggested to check all the features of interest manually so that alterations of the local characteristics of the model are prevented.

The result of the survey activity consists of two digital models [8,53,54,55] that are of paramount relevance in the development of an Integrated Informative System and a Historical Digital Twin of the asset. Indeed, complementary information is recorded and made available to the users, the lack of color data provided by the selected high performance scanning device being well compensated by the photogrammetry that leads to a textured model and offers high-quality orthomosaics of the surveyed object.

From an operational point of view, it is worth noting that the application to the geometrical—scanned—model of the surveyed texture given by the photogrammetry can be performed by using the Geomagic Wrap environment (Colors toolbar) [56] or through the open source software MeshLab [57], as already documented by applications to the historical built heritage [58,59]. This process allows wrapping a 2D/3D textured model to a geometrical model keeping the details of the high-quality starting images.

It should be noted that the photogrammetric model was satisfactory for the purpose of the study, ensuring a good quality 3D texture to the laser scanned model, but a root mean square (RMS) of 1.21 mm resulting from the comparison of the two reconstructed geometrical models (Geomagic Wrap software) pointed out the need of a refinement of the survey procedure to resolve some critical points in the images processing and scaling of the photogrammetric model.

**Figure 9:**
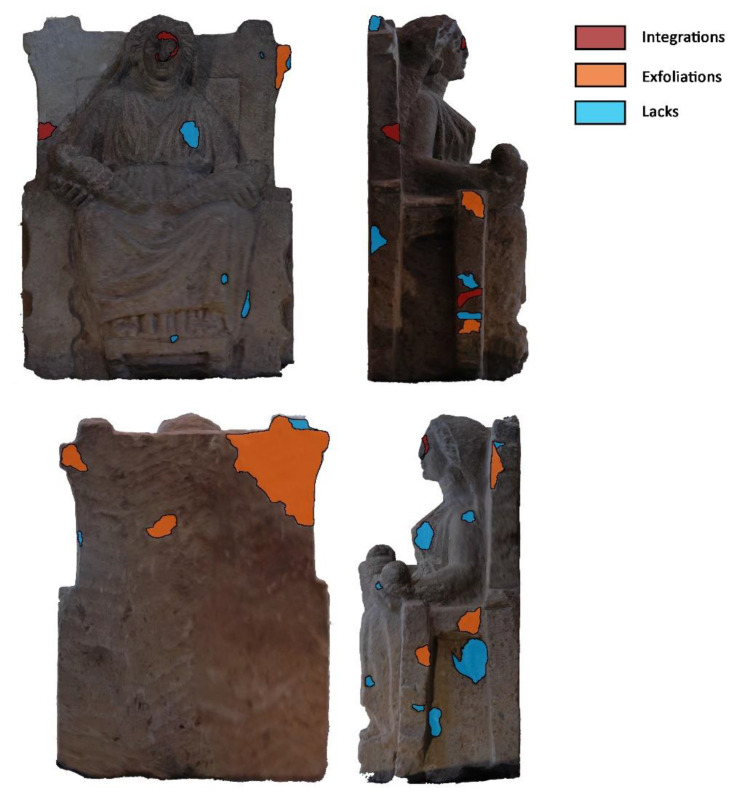
Analysis of degradation forms on the orthomosaics.

However, as the processed orthomosaics demonstrated their reliability in de-scribing the current state of the asset, the refinement of the photogrammetric survey was skipped and the orthomosaics processed in order to perform specific thematic analysis in digital form. Figure 9 shows the above mentioned orthomosaics and outlines the main outcomes of the analysis of the conservation state of the investigated *Mater Matuta*. In particular, the sculpture shows some degradation and alteration forms probably caused by the material properties—the gray tuff is a porous material—and by its permanence under soil deposits. Several missing parts, exfoliations, and chromatic alterations can be detected, both in the main figure and in the throne.

Some stucco work and additions can be observed in the upper part of the throne, in the *Mater*’s left hand, and in the reconstruction of the face, where the sign of the union between the two elements is evident. It is worth noting that the assessment of conservation state benefits from the photogrammetric survey. Indeed, this operation can be repeated over time, recurring also to the crowd sensing systems [60,61], and the results can be easily updated thanks to an operator or through deep learning algorithms [62].

The information acquired was digitized in the SeVAMH protocol, which proved the good state of conservation of artwork and its possible sliding in case of a seismic event. Finally, a modal analysis of the *Mater Matuta* was carried out on the three-dimensional model obtained from laser scanning to assess the dynamic behavior.

**Figure 10:**
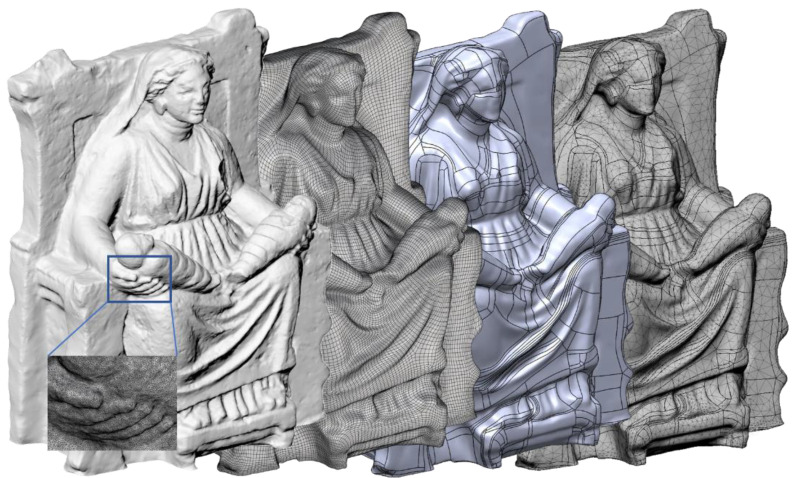
From the very dense scanned and tessellated model to the FE (Finite Element) mesh model, passing through the quad mesh and the surface model (from left to right).

Before running the analysis, an additional post-processing phase of the very dense tessellated model was needed to obtain a FE (Finite Element) model more feasible in the analysis environment (Figure 10). In this phase, starting from the laser-scanned model, a decimated model was firstly generated, as shown in Figure 10 (first on the left). Then, a quad mesh was created as it offers several advantages with respect to triangular mesh. Indeed, with a quad mesh, it is possible to arrange the mesh elements to follow line features of the represented shape.

This characteristic makes it easier to patch the model when generating the surface model, and many advantages also come into rendering and Finite Element Models (FEM) based computational simulations. Quad mesh conversion can be made in several software environments, such as Rhinoceros V7 (by McNeel, Seattle, WA, USA) [63], Blender (by Blender Foundation, Amsterdam, Netherlands) [64], and ReCap Photo (by AutoDesk, San Rafael, CA, USA) [65], or by using a free tool, such as Instant Meshes described in [66] and available in [67]. A regular surface patch model was generated from the quad mesh in the integrated CAD-CAE platform, Fusion 360 (by AutoDesk), by using T-Spline functionalities (a kind of SubD modeling tool [68]) and then converted in a FE model to carry out the modal analysis in the same environment (Figure 10 on the right).

**Figure 11:**
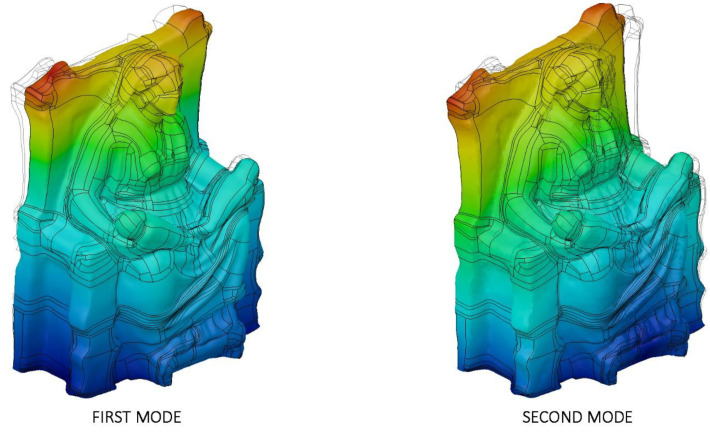
Modal analysis of *Mater Matuta*: first (left—*f*_1_ = 122.2 Hz) and second (left—*f*_2_ = 183.6 Hz) mode; displacement mapping color code ranges between blue (still points) to red (maximum modal displacements).

Results from the simulation highlight that the upper parts of the throne are most susceptible to dynamic action due to the shape and characteristics of the sculpture (Figure 11), but the dimensions lead to very high eigenfrequencies that are far higher than the thresholds, 0.06 s–6.67 Hz, suggested by relevant international seismic codes [69]. As a consequence, the geometrical configuration of *Mater Matuta* (Table 4) makes the sculpture comparable to a single rigid block so that a first approach to the design of interventions aimed at the mitigation of the seismic effects can be made according to the theories of rigid block [22,40,70].

**Figure 12:**
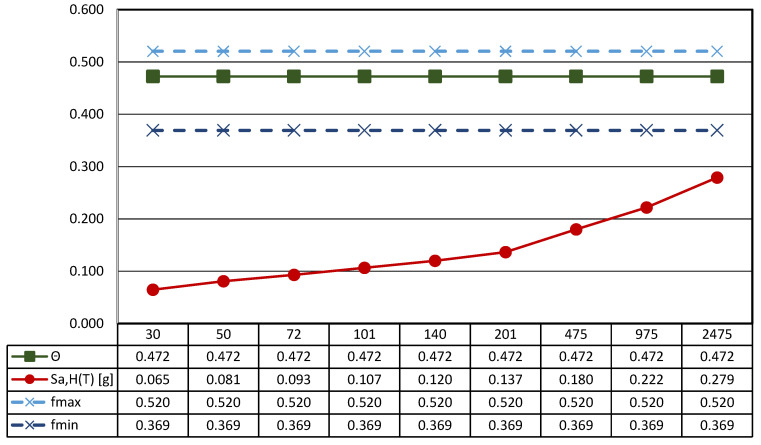
Results of the seismic performance assessment of the *Mater Matuta* under horizontal shaking only.

Figure 12 reports the main results of the seismic characterization of the statue assumed to be placed on the ground and subjected to the horizontal ground shaking described by the Italian design code NTC2018 [48] previously illustrated in Section 3.1.

In more detail, each plot contains four lines: (i) the spectral acceleration at the site for rigid structures (T = 0) identified by a red line; (ii) the function Θ, representative of the horizontal acceleration level that triggers rocking motion in green; and the maximum (iii) and the minimum (iv) acceleration [..] thresholds associated to the sliding of the statue—dashed lines—depending on the maximum and the minimum value of the friction coefficient exhibited by tuff stones. The latter, following a consolidated approach in civil engineering, is estimated as *f* = 2/3 tan*ϕ*, where *ϕ* is the friction angle of the stone [71].

The threshold values are computed through the geometrical data reported in Table 4, where the main characteristics of the base cross-section of the statue (B_min_ and H) and the other geometrical and physical parameters (effective aspect ratio, ϒ, function Θ, and minimum and maximum friction angle, *f_min_* and *f_max_*) derived from the detailed survey process described above are reported.

In more detail, the shape of the base section (z = 0) is allocated in a Cartesian reference (x, y, and z); the location of the center of gravity (x_G_, y_G_, and z_G_), the total mass of the asset, and the centroidal principal axes (ξ and η) of the statue projected on the base plane are reported.

It is easy to recognize that all the thresholds are not exceeded independently from the return period, so it can be argued that the statue can be basically stressed by the dynamic action in the absence of any rigid motion (translation or rotation).

Nonetheless, it is easy to recognize that vertical acceleration is not neutral in the definition of the expected motion of statues. In fact, the spectral vertical acceleration—S_a,V_(T)—reduces the vertical forces acting on the artifact, and the rest condition occurs when:
and *f*g_eff_ ≥ S_a,H_(T) (1)ϒg_eff_ ≥ S_a,H_(T)
where g_eff_ is given by the difference between the gravitational acceleration—g—and the spectral vertical acceleration—S_a,V_(T).

As a consequence, the rest condition can be evaluated considering a reduced friction coefficient—*f*_red_—and a reduced aspect ratio—ϒ_red_—and Equation (1) becomes:
and *f*(g-S_a,V_(T)) ≥ S_a,H_(T) *→*
*f*_red_ ≥ S_a,H_(T) (2)ϒ(g-S_a,V_(T)) ≥ S_a,H_(T) *→* ϒ_red_ ≥ S_a,H_(T)


It is worth noting that reducing the aspect ratio—ϒ_red_—will also reduce the function Θ, which depends on the geometric characteristics of the analyzed artifact. Therefore, the function Θ_red_, when spectral vertical acceleration is considered, is given by:

$$\Theta_{red}=\frac{3\Upsilon_{red}}{4+\Upsilon_{red}^{2}}$$

Therefore, the rocking starts whenever the two conditions in Equation (3) take place:
and *f*_red_ > Θ_red_ (3)S_a,H_(T) > Θ_red_


**Figure 13:**
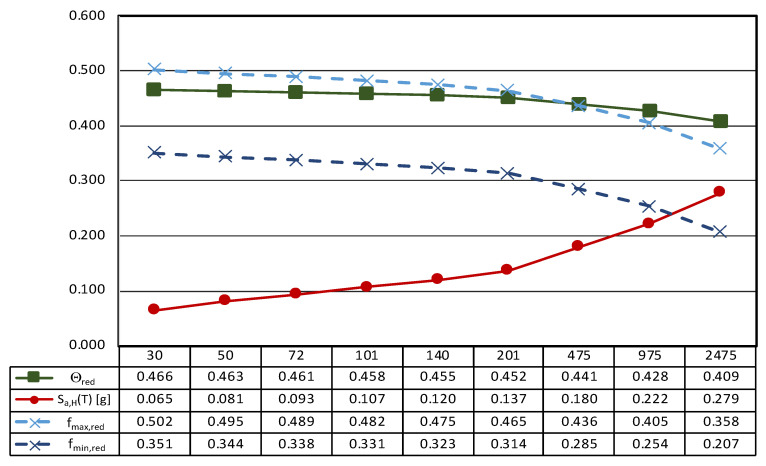
Results of the seismic performance assessment of the *Mater Matuta* under generalized ground shaking.

This issue is addressed in Figure 13, where the thresholds of sliding and rocking motion are computed considering the effects of the spectral vertical acceleration provided by Italian seismic design code [48] for rigid components (T = 0).

It is worth noting that, in the case of generalized ground motion, the thresholds decrease as the seismic intensity increases, in other words moving from frequent to rare events. Consequently, the sliding of the statue seems to be likely in the case of very rare events (T_R_ greater than 975 years) and low values of the friction coefficient. This result shows that, in the case of the *Mater Matuta*, vertical acceleration may play a role in the development of its seismic response, but the return period associated with the generalized ground motion is so high that damage to the asset could come from structural damage to the building (container), whose seismic performance is out of the scope of the work. Conversely, frequent and less rare ground motions should avoid any interference with the asset due to its configuration and geometry.

### 3.3. The Resting Satyr

**Figure 14:**
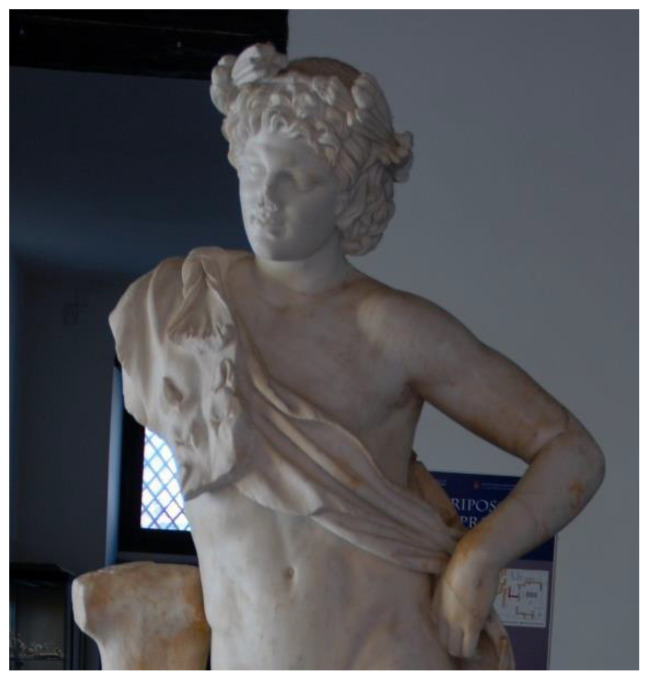
The *Resting Satyr*, details.

The analyses carried out on the bibliographic sources during the historical analysis highlighted that the marble sculpture of *Resting Satyr* (Figure 14) was discovered in the autumn of 2002 during the excavation activities of an ancient Roman villa, a *domus* of imperial age [72]. The *Satyr* is one of the many replicas created in the Adrian–Antonine age, and some technical peculiarities allow us to date the artwork to the first half of the 2nd century A.D, a period of great artistic and building production for the ancient city of Capua.

The *Satyr* is in a frontal position, standing on an oval base and resting against a tree trunk on the right. Similar to other replicas, the *Satyr* has his head raised—slightly rotated to the right—the right shoulder higher than the left and the right leg flexed, rotated outward, and moved back to the left heel [72]. When the sculpture was discovered, it was fragmented and showed several repaired ancient damages. In particular, interventions were aimed at connecting “the based cracked in two parts, the tree trunk to the body of the *Satyr*, the right leg with the left heel and, finally, the pine twig on the right side of the forehead” [72] (pp. 8–9). The connection between the several parts was made with iron bars fixed with lead. These elements caused further damage to the artwork and, in the recent restoration works, steel bars insured with epoxy resin were put in place to connect the many parts in which the sculpture was found, while the lacks were filled with plasterworks aesthetically balanced [72].

The *Resting Satyr* is located on a small and low volume and is housed in one of the first rooms of the museum, introducing the visitor to the exhibition tour and to the knowledge of the history of the city and territory. The metric survey was the result of a scanning laser that required about three hours due to the presence of several details, such as the cape and the wreath around the head, which required more than one scan.

**Figure 15:**
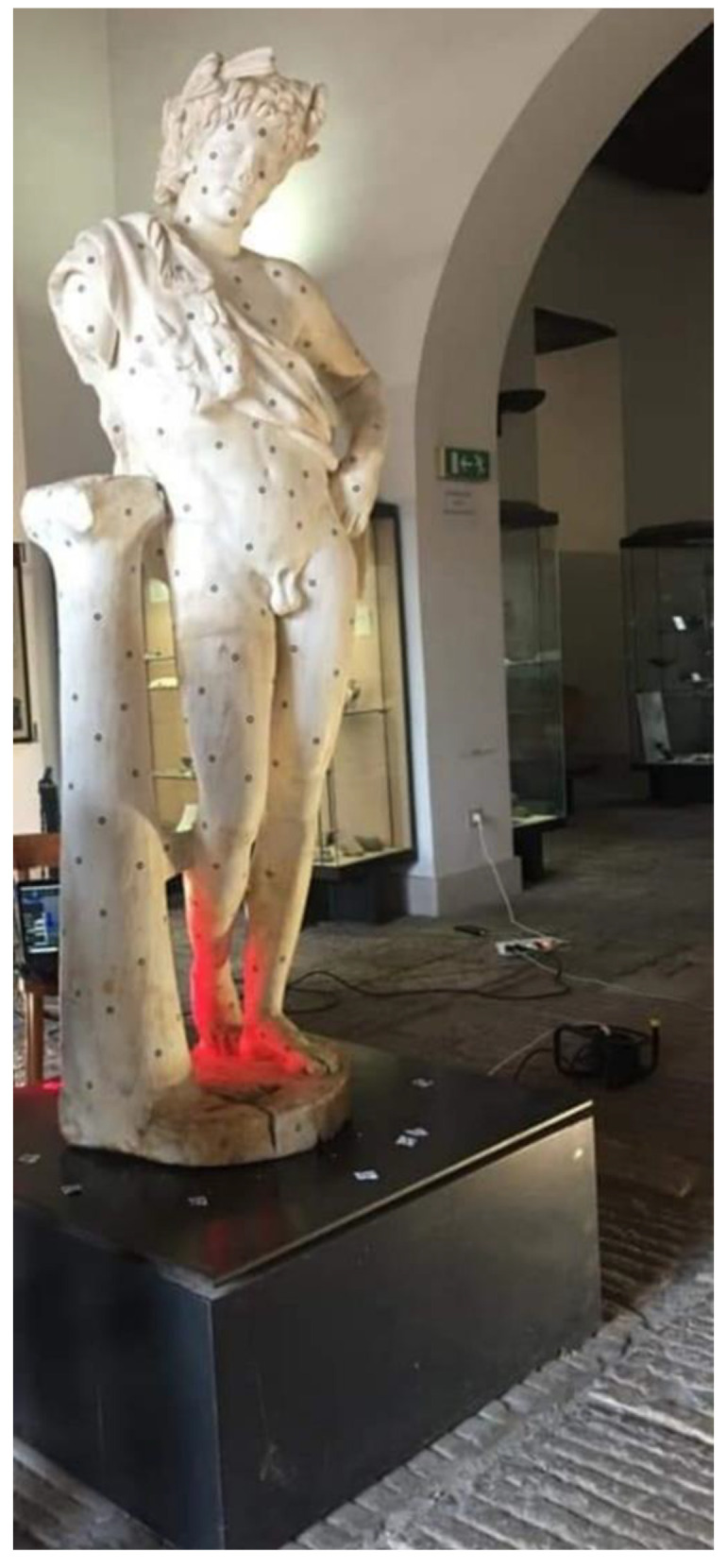
The preliminary phase of the scanning process.

**Figure 16:**
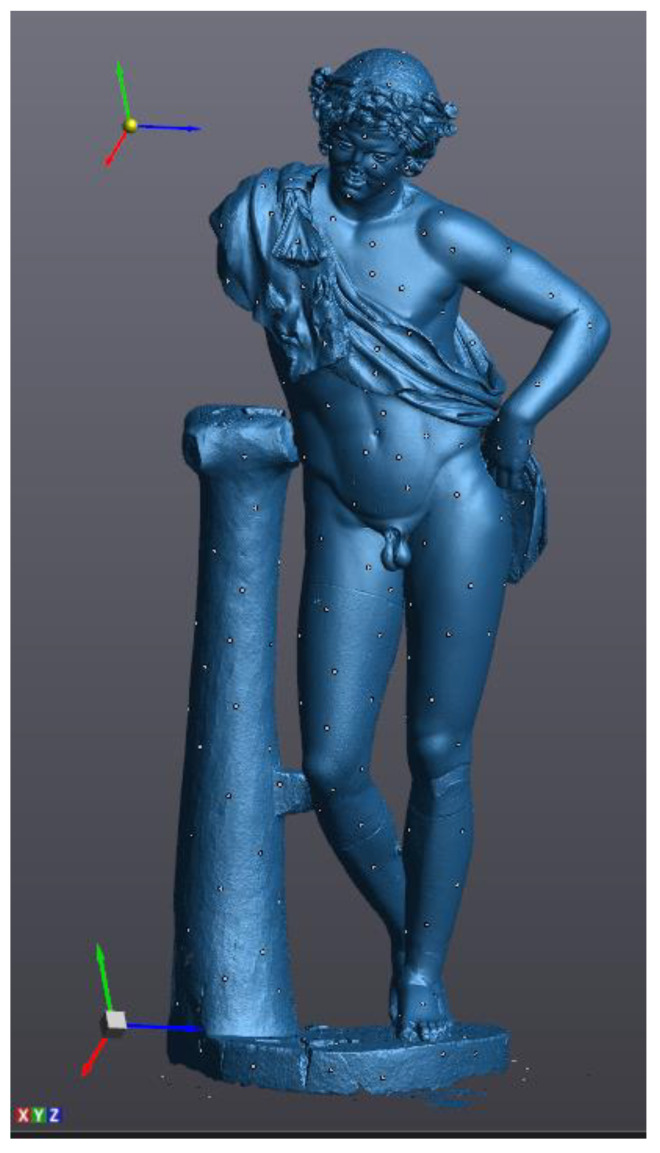
Result of scanning process in VXelements.

About 350 circular markers were put on the statue (Figure 15) at an average distance of 100–200 mm considering the smoothness of the surface. After the scanner calibration, the software setting in VXelements, mainly the automatic optimization of the shutter (to set the best frequency rate of the acquisition, according to the color and reflectance of the surface) and the resolution value (0.05 mm), was carried out. Due to these markers, a network of reference points can be acquired according to the local and global configuration of the surface. Similarly to the *Mater Matuta*, the editing of the model was carried out, resulting in a three-dimensional model characterized by about 5.8 M of vertices and 11.0 M of triangles (Figure 16).

**Figure 17:**
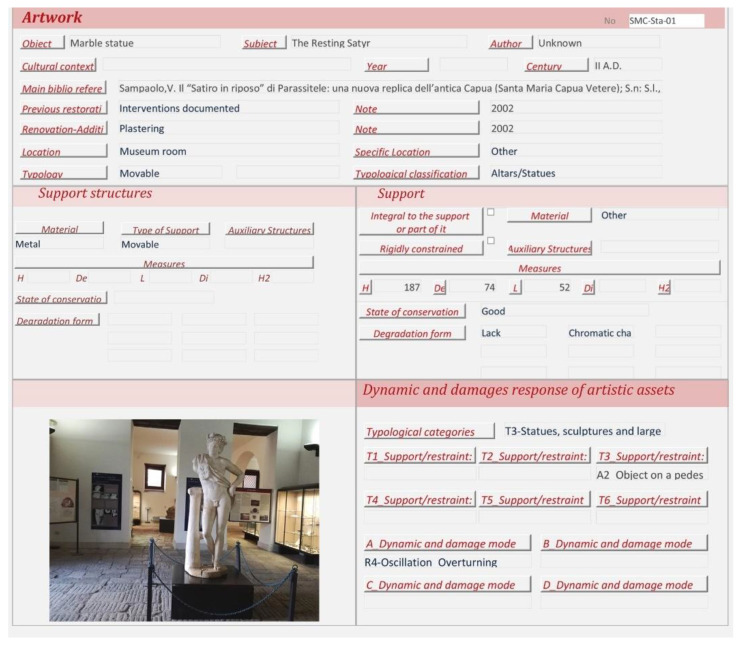
Example of expeditious documentation by SeVAMH data sheet.

**Figure 18:**
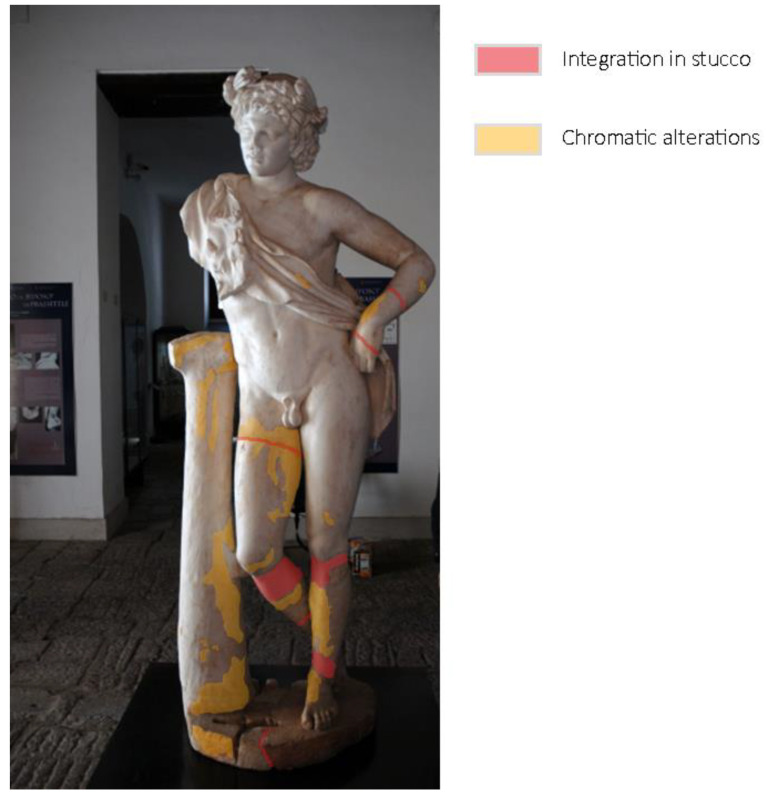
The *Resting Satyr*, current state, and alteration form.

At the same time, the SeVAMH protocol was applied to catalog the information derived from bibliographic sources and acquired during the survey campaign. The state of conservation of the statue, its seismic response, and damage mechanisms have been assessed from a qualitative point of view through the information acquired in the SeVAMH form (Figure 17). Currently, the *Satyr* has a good state of conservation, despite the several defects caused by past damage and the additions gathered by the recent restoration work. The base of the statue, the tree trunk, and the *Satyr*’s legs show some chromatic alterations, probably caused by its permanence under debris and other materials (Figure 18).

The detected alterations do not affect the statue’s preservation; however, the lack of anti-seismic devices and suitable support elements could threaten its safety in case of an earthquake. Indeed, the *Satyr* could oscillate or slide under seismic action. Therefore, a high-intensity seismic event could lead to the statue’s overturning and damage in several parts, causing the loss of this valuable artifact.

The three-dimensional model resulting from laser scanning was further processed to perform detailed analyses and evaluate the structural behavior. The result of the post-processing phase is a surface model more manageable during the simulation phase as it requires lower calculation effort and shorter processing time. VXelements was used to edit mesh by sampling data, filling holes, reducing scanning noise, and, finally, completing the three-dimensional reconstruction with the creation of the waterproof closed mesh model.

**Figure 19:**
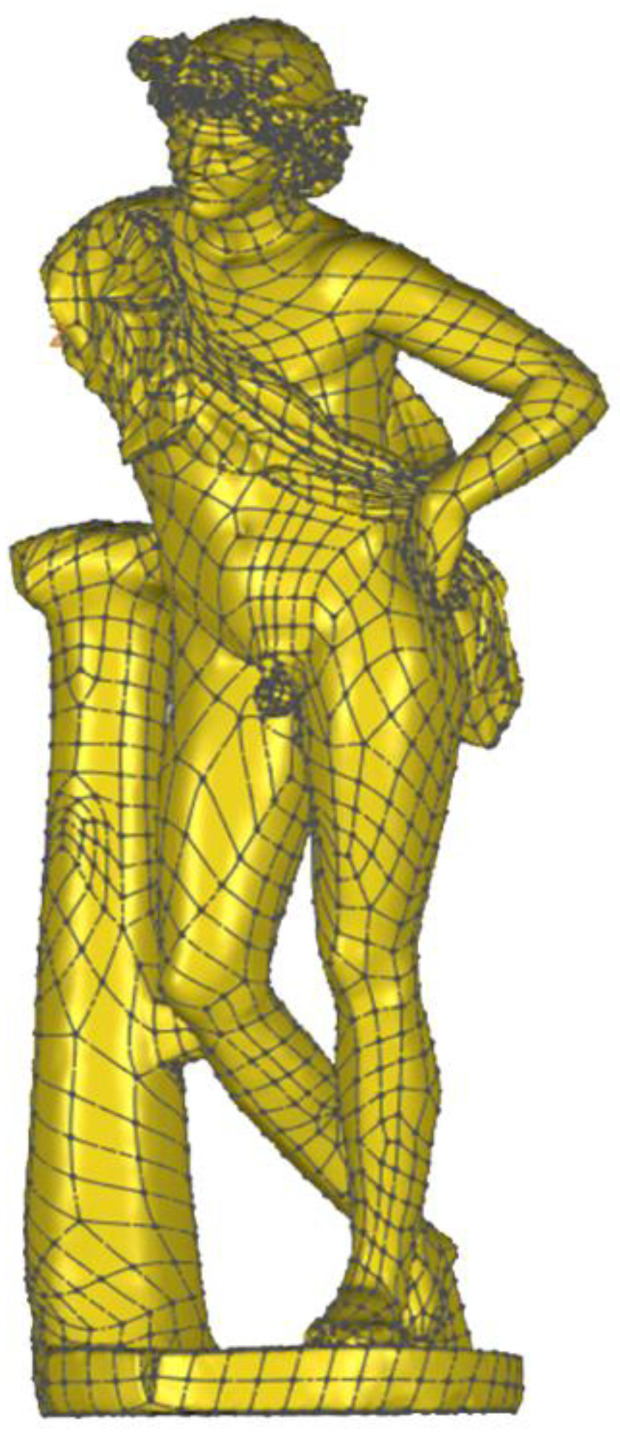
Solid model of the *Resting Satyr*.

During this scanning process, an input filter was applied to data acquired through laser scanning thanks to the smoothed nature of many parts of the statue. The final result of the mesh editing process is a mesh model characterized by about 400 k triangular polygons, then best fitted by about 4.2 k faces, with higher density where more details occur (Figure 19).

The last phase of the workflow is associated with the structural characterization of the asset, using global and local properties. Indeed, the three-dimensional geometrical model—even if not yet optimized due to the lack of geometrical and material details of the reassembling process—can be used to accomplish structural analysis of the sculpture, considering also its behavior as a rigid block [21], and collect useful information related to the sensitivity of the asset to external loads.

Therefore, the model of the *Satyr* was imported via Step file into Fusion 360 software to perform the modal analysis and the static stress one (Von Mises). Both structural analyses were carried out in the simplified assumption of homogeneous material and lack of damage. Indeed, the stucco integrations and the steel bars placed during the last restoration works were not considered.

**Figure 20:**
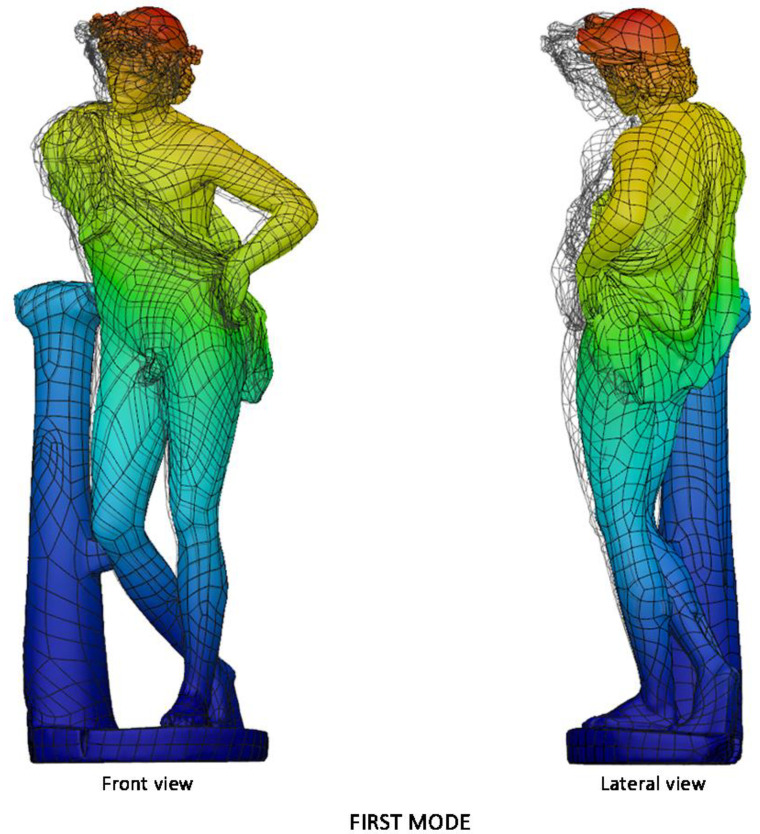

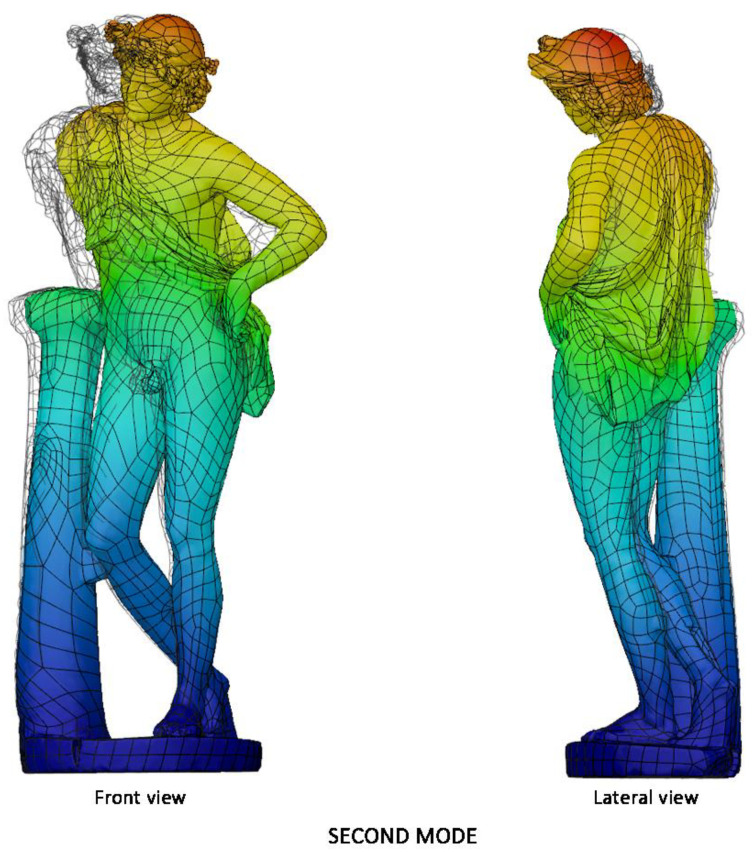
Modal analysis of *Resting Satyr*: first (top—*f*_1_ = 20.7 Hz) and second (bottom—*f*_2_ = 46.5 Hz) mode; displacement mapping color code ranges between blue (still points) to red (maximum modal displacements).

Figure 20 collects the pictures of the first and the second modal shapes of the *Resting Satyr* computed by the modal analysis, as already mentioned, of the undamaged and homogeneous 3D FE model of the asset. Two viewpoints are reported for each modal shape, associated with the first modal frequency *f*_1_ =20.7 Hz and the second one *f*_2_ = 46.5 Hz.

The slender shape of the *Resting Satyr*, compared with the *Mater Matuta*, is demonstrated by the lower value of the first frequency that is just beyond the limit—16.67 Hz—of the rigid non-structural components after ASCE-07 provisions [69], so that again the statue can be classified as a rigid, seismic sensitive structure.

On this point, it is worth noting that the result is not reliable as the one achieved for the *Mater Matuta*; this is due to the simplified assumptions of the modal analysis, which does not take account of the discontinuities generated by the past fractures of the statue and the effects of the reconstructive restoration performed in recent times. A more detailed analysis of the asset could be carried out using a more refined FE model, but more reliably and efficiently by means of a dynamic identification of the statue carried out through operational modal analyses (OMA), which do not need any excitation, and can be carried out by recording the environmental vibrations of the asset [73].

Table 5 reports the main geometrical characteristics of the statue retrieved by the geometrical model of the asset and associated with the rigid body subjected to base excitations [22,40,74], on the analogy with the data reported in Table 4 for the *Mater Matuta*.

It is worth noting that the geometrical analysis of the model, namely the shape of the base section, the location of the center of gravity, the total mass of the asset, and the centroidal principal axes of the statue, offers to the seismic analysts reliable, accurate, and manageable data in line with the SeVAMH protocol.

The friction coefficient (*f*) of the *Satyr*, as for the *Mater Matuta*, was defined by using the value of the friction angle reported in the literature [75] and properly reduced according to the approaches used in civil engineering (*f* = 2/3 tan*ϕ*, where *ϕ* is the friction angle).

**Figure 21:**
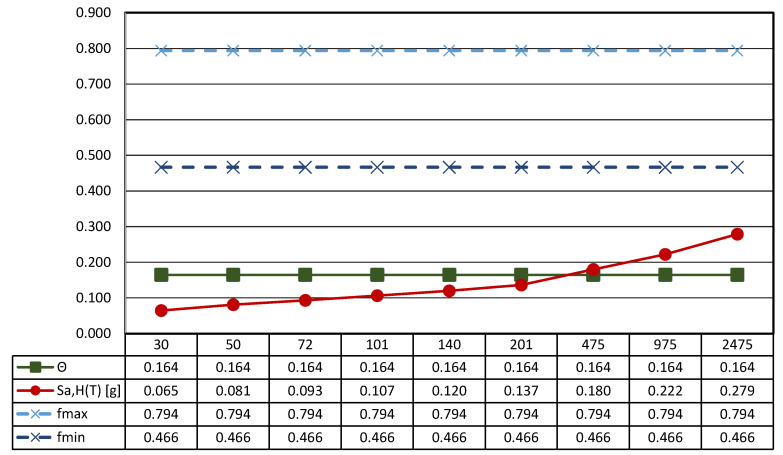
Results of the seismic performance assessment of the *Resting Satyr* under horizontal shaking only.

**Figure 22:**
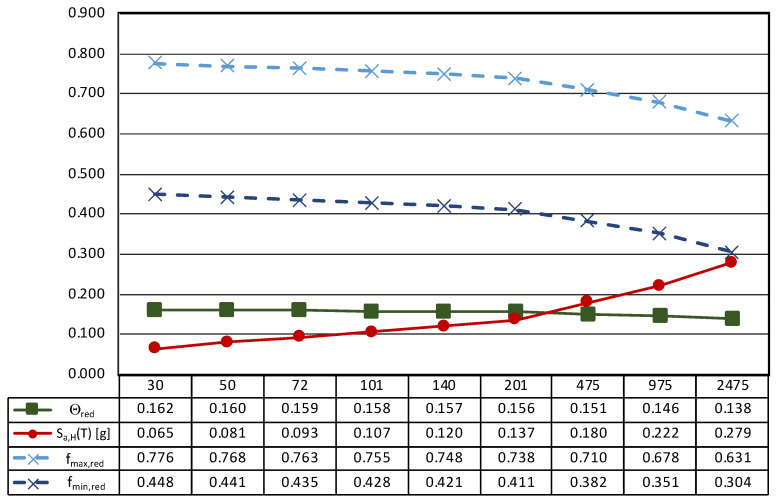
Results of the seismic performance assessment of the *Resting Satyr* under generalized ground shaking.

The sculpture has an aspect ratio (ϒ) that makes it a slender structure; furthermore, considering that the aspect ratio is smaller than the friction coefficient (Figure 21 and Figure 22), the statue will be susceptible to rocking motion [70,74].

The static stress analysis performed on the same simplified model was useful to assess the feasibility and reliability of the workflow; this analysis is the basic step to performing a detailed analysis of artistic asset and to identify the critical areas.

**Figure 23:**
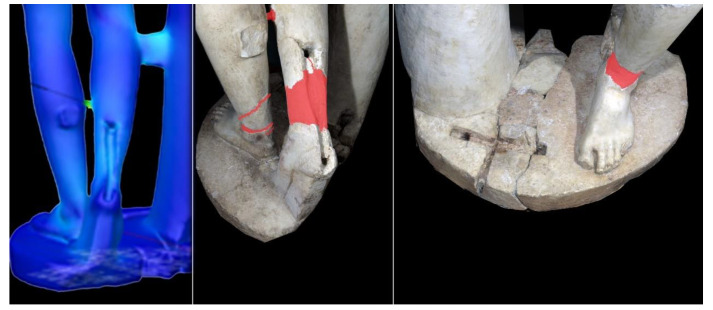
Results of static stress analysis versus previous restoration works.

The results highlight that, due to the shape and current configuration of the *Satyr*, stress concentrations are located at the connections of the sculpture to the base and to the tree trunk. In other words, the parts of the sculpture repaired in the past are critical in bearing the loads and need to be further investigated (Figure 23). This is an important outcome of the analysis, as it guides the operators in defining the key zones and details of the assets that need to be investigated in detail. In such a way, all relevant geometrical, material, and interface properties of the installed mechanical devices could be assessed and monitored in time by means of appropriate non-destructive techniques [76,77,78].

This additional diagnostic phase, indeed, appears to be propaedeutic for the development of a detailed structural analysis on a model incorporating heterogeneous materials and the steel bars located in the left leg, in the right leg to connect it with the heel, in the connection between the trunk and the right leg, and finally all the relevant interface response of the mechanical anchors installed during the reconstruction process, moving from the connecting element between the two legs [72].

The performed analysis and the achieved results represent a useful reference to planning an experimental analysis campaign to optimize the mechanical model and the structural analysis. In this way, several tools designed to support the conservation and management demands of artistic assets exposed in seismic areas could be strengthened.

## 4. Conclusions

The safety and safeguard of cultural heritage, in particular of artistic heritage, are complex tasks that require an interdisciplinary, simultaneous, and joint effort between technical and humanistic professionals. Such operations can be facilitated by developing effective digital solutions for relevant data acquisition, processing, and reporting in a way that the professionals involved in the process have correct and updated information.

The present paper extended and consolidated some aspects of an interdisciplinary study reported in [29], by discussing the methodological framework behind the development of the static data associated with IISs and exploring the use and integration of digital survey tools in view of a comprehensive application of the HDT paradigm. The assessment of the digital process was carried out with reference to two extraordinary artworks exhibited within the Archaeological Museum of Ancient Capua. In particular, the combination of a high-precision laser-scanned three-dimensional model, a high-quality orthomosaics for the assessment of conservation state, and other relevant data gathering according to an available survey protocol (SeVAMH), confirmed the advantages associated with the proposed interdisciplinary workflow. The latter, indeed, integrates traditional approaches with those based on digital technologies in the assessment of the current state of artistic heritage leading to relevant benefits in the definition of conservation actions for these artifacts due to the capability of the digital flow of generating structural models and mechanical performance data suitable for the design and management of the sites and structures hosting the moveable assets.

The data collected and the results achieved in the validation of some fundamental steps of the proposed integrated procedure are encouraging, but need further efforts and investigations for generalized practical applications. Moreover, the results obtained show that IIS and HDT may afford the correlation between digital replicas of cultural heritage to other databases or web platforms, finalizing new procedures based on the automation of data integration and analysis. Therefore, optimal strategies and tools for the conservation, maintenance, and protection of historical artifacts and valuable assets can be defined by expanding the capabilities of present IISs, pushing them towards fully operational HDTs.

## Figures and Tables

**Table 1:** Data acquired from interdisciplinary survey.

Main Phase	Post-Processing	Type of Data
Historical Analysis	Catalogue of historical modifications	Qualitative data
Dimensional Analysis	Two-dimensional representation (plans, elevations, sections) of the artefact Three-dimensional representation (digital model)	Metric data
Metrical Characterization	Catalogue of materials and construction techniques Mechanical parameters of the materials	Qualitative data Quantitative data
Condition Assessment	Catalogue of degradation and damage Natural frequencies, modal shapes, …	Qualitative data Quantitative data

**Table 2:** Summary of photogrammetric acquisition parameters.

Camera	Nikon D810
Focal length	34 mm
Image overlap	> 80%
Distances from object	⋍1200 mm
Shooting heights (measured from the base of the statue)	Lower level: ⋍235 mm Medium level: ⋍820 mm Upper level: ⋍1690 mm
Shooting angles (with respect to vertical plane)	Lower level: ⋍0° Medium level: ⋍15° Upper level: ⋍35°
GDS—Ground Sample Distance	0.2 mm/pixel

**Table 3:** Summary of the seismic hazard parameters at the site, Italian NTC 2018.

T_R_ (y)	a_g,H_ (g)	F_0_	T_C_ * (s)	S_a,H_(T = 0) (g)	S_a,V_(T = 0) (g)
30	0.043	2.379	0.285	0.065	0.018
50	0.054	2.372	0.321	0.081	0.026
72	0.062	2.421	0.335	0.093	0.031
101	0.071	2.430	0.350	0.107	0.039
140	0.080	2.469	0.362	0.120	0.046
201	0.091	2.489	0.375	0.137	0.056
475	0.120	2.526	0.426	0.181	0.085
975	0.148	2.605	0.446	0.221	0.115
2475	0.186	2.721	0.484	0.280	0.163

**Table 4:** Summary of the main geometric features of the asset for seismic rigid block analyses.

Shape of Base Section		Center of Gravity (mm)			Mass (kN∙s^2^/m)	Centroid Principal Axes	
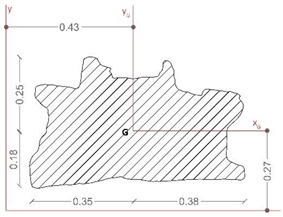		x_G_	y_G_	z_G_		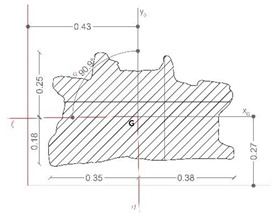	
		447	264	367	0.202		
B_min_ (m)	H (m)	Υ=BminH (g)		Θ=3ϒ(4+ϒ2) (g)		*f_min_*	*f_max_*
0.260	0.367	0.709		0.472		0.369	0.520

**Table 5:** Summary of the key characteristics of the assets for seismic rigid block.

Shape of Base Section		Center of Gravity (mm)			Mass (kN∙s^2^/m)	Centroid Principal Axes	
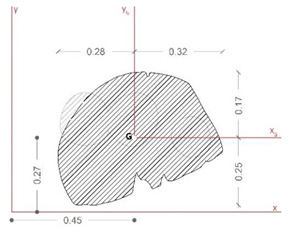		x_G_	y_G_	z_G_		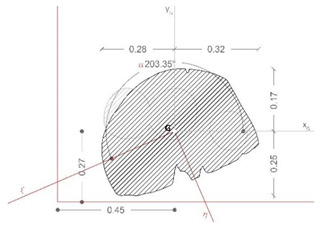	
		454	297	902	0.365		
B_min_ (m)	H (m)	ϒ = B_min_/H		$\Theta =\frac{3ϒ}{4+{ϒ}^{2}}$		*f_min_*	*f_max_*
0.260	0.367	0.709		0.472		0.466	0.794

## Data Availability

Not applicable.
